# Acknowledgement of manuscript reviewers 2018

**DOI:** 10.18332/tid/104388

**Published:** 2019-02-13

**Authors:** James Elliott Scott, Israel Agaku

**Affiliations:** 1 Department of Oral Biology, Rady Faculty of Health Sciences, University of Manitoba, Winnipeg, Canada; 2 Biology of Breathing Group, Children’s Hospital Research Institute, University of Manitoba, Winnipeg, Canada; 3 Harvard School of Dental Medicine, Boston, United States

## CONTRIBUTING REVIEWERS

The editors of Tobacco Induced Diseases would like to thank all our reviewers who have contributed to the journal in Volume 16 (2018).

**Antonio Abbate**

Italy

**Suhaj Abdulsalim**

Saudi Arabia

**Aderonke Akinkugbe**

United States

**Nour Al-Sawalha**

Jordan

**Abdulmohsen Al-Zalabani**

Saudi Arabia

**Muath Aldosari**

United States

**Fatmah R. Ali**

United States

**Qasem Alomari**

Kuwait

**Hemantha Amarasinghe**

Sri Lanka

**Tatiana Andreeva**

Ukraine

**José Antunes**

Brazil

**René Arrazola**

United States

**Rehab Auf**

United States

**Francisco Ayesta**

Spain

**Raed Bahelah**

Yemen

**Sabeeh A. Baig**

United States

**Montse Ballbè**

Spain

**Davut Baltaci**

Turkey

**Amitav Banerjee**

India

**Yael Bar-Zeev**

Australia

**Arzu Beklen**

Turkey

**Helen Binns**

United States

**Linda Bledsoe**

United States

**Pinar Bostan**

Turkey

**Marc T. Braverman**

United States

**Leslie Ann Brick**

United States

**Betsy Brock**

United States

**Jennifer Brown**

United States

**Nurcan Buduneli**

Turkey

**Karen Calabro**

United States

**Priscilla Callahan-Lyon**

United States

**Natalie Capps**

United States

**Ralph Caraballo**

United States

**Victor Cardenas**

United States

**Timothy Chambers**

New Zealand

**Po-Yin Chang**

United States

**Omar Chehab**

Lebanon

**Julia Chen**

United States

**Onyema Chido-Amajuoyi**

United States

**Parimal Chowdhury**

United States

**Fatma Cihan**

Turkey

**Nursan Cinar**

Turkey

**Bradley Collins**

United States

**Mark Conner**

United Kingdom

**Nancy Tess Boley Cruz**

United States

**Dardo Curti**

Uruguay

**Nazir A. Dar**

India

**Mike Daube**

Australia

**Bertrand Dautzenberg**

France

**Richard Daynard**

United States

**Silvio De Flora**

Italy

**Dennis de Ruijter**

Netherlands

**Olufemi Olumuyiwa Desalu**

Nigeria

**Ligia Devóglio**

Brazil

**Yuan Di**

Australia

**Amanda Dickinson**

United States

**Jeremy E. Drehmer**

United States

**Michaela Dušková**

Czech Republic

**Sarah Edwards**

Canada

**Marwan E. El-Sabban**

Lebanon

**Ubiracé Fernando Elihimas Jr**

Brazil

**Edward Ellerbeck**

United States

**Lucinda England**

United States

**Mukremin Er**

Turkey

**Ebru Erdemir**

Turkey

**Nazmiye Erdogan**

Turkey

**Daniel Erku**

Ethiopia

**Patricia Escobedo**

United States

**William Evans**

United States

**Changyong Feng**

United States

**Filippos Filippidis**

United Kingdom

**Elizabeth Flott**

United States

**Omid Fotuhi**

Canada

**Diane Francis**

United States

**Kate Frazer**

Ireland

**Deborah Fritz**

United States

**Erika Froelicher**

United States

**Silvia Fustinoni**

Italy

**Silvano Gallus**

Italy

**Esther Garcia-Esquinas**

Spain

**Gladwell Gathecha**

Kenya

**Patrick Geraghty**

United States

**Wan Ghani**

Malaysia

**Laura Gibson**

United States

**Alexander Gilkes**

United Kingdom

**Gary A. Giovino**

United States

**Charis Girvalaki**

Greece

**Rebecca Glover-Kudon**

United States

**Tatiana Goerig**

Germany

**Giuseppe Gorini**

Italy

**Trisha Greenhalgh**

United Kingdom

**Osman Günay**

Turkey

**Hakan Gunen**

Turkey

**Prakash Gupta**

India

**Joseph Guydish**

United States

**Takashi Hanioka**

Japan

**Wojciech Hanke**

Poland

**Alyssa Harlow**

United States

**Arusyak Harutyunyan**

Armenia

**Linnea Hedman**

Sweden

**Lisa Henriksen**

United States

**Gholamreza Heydari**

Iran

**Heikki Hiilamo**

Finland

**Rosemary Hiscock**

United Kingdom

**Sai Yin Ho**

Hong Kong

**David Homa**

United States

**Melbourne Hovell**

Israel

**Pei-Ting Hsu**

Taiwan

**Hsien-Liang Huang**

Taiwan

**Ruijie Huang**

China

**Karin Hummel**

Netherlands

**Muhammad Jami Husain**

United States

**Azmina Hussain**

Pakistan

**Sinem Iliaz**

Turkey

**Farhad Islami**

United States

**Ali Jawaid**

Pakistan

**Sun Jee**

Republic of Korea

**Nan Jiang**

United States

**Kevin John**

United States

**Nitin Joseph**

India

**Ibrahim Ali Kabbash**

Egypt

**Steven Ndugwa Kabwama**

Uganda

**Sara Kalkhoran**

United States

**Gordana Kamceva**

FYROM

**Dilek Karadoğan**

Turkey

**Kamaleddin Karimyan**

Iran

**Kota Katanoda**

Japan

**Erinne Kennedy**

United States

**Georges Khalil**

United States

**Tamkeen Khan**

United States

**Joshua Kibet**

Kenya

**Daehyun Kim**

Republic of Korea

**Leon Kosmider**

United States

**Susi Kristina**

Indonesia

**Allison Kurti**

United States

**Sang Haak Lee**

Republic of Korea

**Sungkyu Lee**

Republic of Korea

**Yo Lee**

Republic of Korea

**Joshua B. Lewis**

United States

**Alex Liber**

United States

**Adam Lippert**

United States

**Feng Liu**

China

**Xiaoqiu Liu**

Italy

**Huai Loh**

Malaysia

**Tran Long**

Australia

**Brett Loomis**

United States

**Ángel López-Nicolás**

Spain

**Lucia Maria Lotrean**

Romania

**Liya Lu**

United Kingdom

**Mark Lucherini**

United Kingdom

**Ingeborg Lund**

Norway

**Dan Luo**

China

**James Macinko**

United States

**Renee Magnan**

United States

**Jeanne Mahoney**

United States

**Hadii Mamudu**

United States

**Michelle Manderski**

United States

**Lamberto Manzoli**

Italy

**Cristina Martínez**

Spain

**Kristy Marynak**

United States

**Mohammad Masjedi**

Iran

**Charu Mathur**

United States

**Yuuki Matsumoto**

Japan

**Debra McCallum**

United States

**Sam McCrabb**

Australia

**Anne Melzer**

United States

**Gülengül Mermer**

Turkey

**Alipasha Meysamie**

Iran

**Stephen Miller**

United States

**Yoshiaki Minakata**

Japan

**Francis Mitrou**

Australia

**Laurenţiu Mogoantă**

Romania

**Jawad Mohammed**

United Kingdom

**Kahee Agid Mohammed**

United States

**Surapaneni Mohan**

India

**Ute Mons**

Germany

**Annie Montreuil**

Canada

**Meghan Moran**

United States

**Narine Movsisyan**

Czech Republic

**Donna Anne Murnaghan**

Canada

**Joshua Muscat**

United States

**Mark G. Myers**

United States

**Rima T. Nakkash**

Lebanon

**Jevae Nelson**

United States

**Kenneth Bruce Newbold**

Canada

**Nhung Nguyen**

Vietnam

**Fernando Nogueira**

Brazil

**Margaret Nolan**

United States

**Fariz Nurwidya**

Japan

**Satomi Odani**

United States

**Masaki Ohsawa**

Japan

**M.A.A. Oliveira Serra**

Brazil

**Sophie Orton**

United Kingdom

**Ellis Owusu-Dabo**

Ghana

**Pannee Pantaewan**

Thailand

**R. Steven Pappas**

United States

**Linda Pederson**

United States

**Nasheeta Peer**

South Africa

**Melinda Pénzes**

Hungary

**Michele Pergadia**

United States

**Irene Pericot-Valverde**

United States

**Armando Peruga**

Chile

**Andrew Pipe**

Canada

**Barbara Pizacani**

United States

**Kinga Polańska**

Poland

**Yayi Prabandari**

Indonesia

**Elena Raffetti**

Italy

**Deepa Raghavan**

United States

**Angela Ratsch**

Australia

**Sofia Ravara**

Portugal

**Martin Raw**

United States

**Mirjam Reutter**

Germany

**Luz Reynales Shigematsu**

Mexico

**Polosa Ricardo**

Italy

**Megan E. Roberts**

United States

**Paul Roman**

United States

**Daniel Romer**

United States

**Craig Ross**

United States

**Hana Ross**

South Africa

**Renata Rossi e Silva**

Brazil

**Kathleen Ruff**

Canada

**Tobias Rüther**

Germany

**Maguy Saffouh El Hajj**

Qatar

**Banu Salepci**

Turkey

**Jonathan Samet**

United States

**Rassamee Sangthong**

Thailand

**Kazunari Satomura**

Japan

**Benjamin Schüz**

Germany

**David Scott**

United States

**Arielle Selya**

United States

**Saul Shiffman**

United States

**Carlos Sillero**

United Kingdom

**Akansha Singh**

India

**Pranil Man Singh Pradhan**

India

**Nithat Sirichotiratana**

Thailand

**Nina Skavlan Godtfredsen**

Denmark

**Danielle Smith**

United States

**Matiwos Soboka**

Ethiopia

**Chandrashekhar Sreeramareddy**

Malaysia

**Gerry Stimson**

United Kingdom

**Elizabeth Stuyt**

United States

**Hua Su**

United States

**Walton Sumner**

United States

**Steve Sussman**

United States

**Girija Syamlal**

United States

**Chie Taniguchi**

Japan

**Mehmet Tecellioglu**

Turkey

**Mary Thompson**

Canada

**Steven Thomsen**

United States

**Philip Tonnesen**

Denmark

**Serena Tonstad**

Norway

**Antigona C. Trofor**

Romania

**Panagiotis N. Tsikouras**

Greece

**Anna Tzortzi**

Greece

**Bukola Usidame**

United States

**Juliana Uzeloto**

Brazil

**Eva Anne Marije van Eerd**

Netherlands

**Paul Vanderkam**

France

**Tord Vedøy**

Norway

**Michelangelo Vestita**

Italy

**Matteo Vitali**

Italy

**Paulo Vitória**

Portugal

**Nicholas J. Wagner**

United States

**Kelvin Man Ping Wang**

Hong Kong

**Pingman Wang**

Hong Kong

**Yingning Wang**

United States

**Kenneth D. Ward**

United States

**Kenneth Warner**

United States

**Lynn Weber**

Canada

**David A. Welsh**

United States

**Jean Wong**

Canada

**Biao Xu**

China

**Gonghuan Yang**

China

**Ying Yang**

China

**Xiao-Hua Ye**

China

**Selen Yegenoglu**

Turkey

**Shaoman Yin**

United States

**Emilia Zainal Abidin**

Malaysia

**Fengyu Zhang**

United States

**Li Zhou**

China

**Wioleta Zielińska-Danch**

Poland

